# Prehabilitation Program in Elderly Patients: A Prospective Cohort Study of Patients Followed Up Postoperatively for Up to 6 Months

**DOI:** 10.3390/jcm10194500

**Published:** 2021-09-29

**Authors:** Claire Malot, Astrid Durand-Bouteau, Nicolas Barizien, Antoine Bizard, Titouan Kennel, Marc Fischler, Enrico Minnella, Morgan Le Guen

**Affiliations:** 1 Department of Sport Medicine and Readaptation, Hôpital Foch, 92150 Suresnes, France; malot.claire@gmail.com (C.M.); n.barizien@hopital-foch.com (N.B.); 2 Department of Anesthesiology, Hôpital Foch, 92150 Suresnes, France; astridbouteau@gmail.com (A.D.-B.); m.leguen@hopital-foch.com (M.L.G.); 3 Mobile Unit of Acute Geriatrics, Hôpital Foch, 92150 Suresnes, France; a.bizard@hopital-foch.com; 4 Department of Research and Innovation, Hôpital Foch, 92150 Suresnes, France; t.kennel@hopital-foch.com; 5 Department of Anesthesia, Montreal General Hospital, McGill University Health Centre, Montreal, QC H4A 3J1, Canada; minnella.enrico@hsr.it; 6 UFR Simone Veil, Université Versailles-Saint-Quentin-en-Yvelines, 78000 Versailles, France

**Keywords:** prehabilitation, elderly, surgery, walk test

## Abstract

The preoperative period may be an opportune period to optimize patients’ physical condition with a multimodal preoperative program. The impact of a “prehabilitation” program on elderly patients is discussed. This mono-center observational cohort study included consecutively 139 patients planned for major abdominal and thoracic surgery, with 44 in the control group (age < 65) and 95 in the elderly group (age > 65). All patients followed a “prehabilitation” program including exercise training, nutritional optimization, psychological support, and behavioral change. Seventeen patients in the control group and 45 in the elderly group completed the study at six months. The 6-minute walk test (6 MWT) increased in both groups from the initial evaluation to the last (median value of 80 m (interquartile range 51) for those under 65 years; 59 m (34) for the elderly group; *p* = 0.114). The 6 MWT was also similar after one month of prehabilitation for both populations. The rate of postoperative complications was similar in the two groups. Prehabilitation showed equivalence in patients over 65 years of age compared to younger patients in terms of increase in functional capabilities and of postoperative evolution. This multimodal program represents a bundle of care that can benefit a frailer population.

## 1. Introduction

According to different international surveys in the surgical field, about one-quarter of patients over 65 years old will require specific management of perioperative care in future years [1]. Surgery on the elderly leads to an elevated risk of a range of postoperative complications [2,3]. These include mainly postoperative delirium, pulmonary infection, cardiovascular events, and an overall higher rate of postoperative morbidity, extended length of stay, and/or mortality. They also saw a general long-term decline in health, cognition, functional status, and quality of life after surgery [4,5]. In addition, immediate postoperative complications can result in and amplify a long-term decline of health and functional independence. 

Geriatricians have recently developed a standardized phenotype of the elderly from the description by Rockwood et al. concerning the number of functional defects reflecting the loss in the functional reserve [6]. A patient is qualified as robust (successful aging in good health), normally aging (pre-frail), and pathological (frail) with a high risk of dependency in the last category. A stressor or an unplanned event such as an infection or a surgery may severely impact the patient’s outcome with a risk of loss of autonomy depending on the phenotype [7]. The perioperative challenge remains to bring a global medical approach as early as the preoperative visit to limit unfavorable outcomes and promote an evolution towards a change in the functional status of the elderly [8]. Despite an event-free postoperative period, the perioperative period is associated with a 20% to 40% decrease in physical and functional abilities, and this reversible negative impact may be attenuated through postoperative programs such as rehabilitation. However, in every case, it takes several months to recover the preoperative “physiological” status, [4] and the postoperative rehabilitation or enhanced recovery after surgery (ERAS) program may be limited in this population [9]. It, therefore, makes sense to try to improve the physical fitness of patients even before the surgery is finished. By increasing the fitness level of frail patients, they could stock up on more reserves [10].

As demonstrated by Carli et al., the preoperative period may be an opportune time to optimize the patients’ physical condition with a multimodal preoperative program named “prehabilitation” [11]. Prehabilitation aims to hasten functional recovery and reduce perioperative complications using supervised endurance exercise training, promotion of physical activity, nutritional therapy, and anxiety-reduction intervention [12]. Many care-providers (e.g., dietitians, physiotherapists, physicians, etc.) are mobilized to better prepare patients for an upcoming procedure with the application of “patient-centered care” [11]. This is included in a broader concept of perioperative care. Recently, Minnella et al. demonstrated a maximal efficiency of this program among patients with poor reserves, such as elderly patients [13].

Our hypothesis was that the impact of prehabilitation is similar in young patients (<65 years old) and in elderly patients (>65 years old), despite a high risk of decreased functional reserve. This threshold was given by the World Health Organization (WHO) and is thought to be the limit worldwide, regardless of the main social reference. 

## 2. Materials and Methods

This retrospective study, performed in a nonprofit tertiary care hospital, was approved on 4 April 2016, by the Ethical Committee of the French Society of Anesthesiology and Critical Care (IRB 00010524-2016-017). Patients were informed that their data could be used for research purposes and were given the opportunity to refuse. 

### 2.1. Study Population

From January 2017 to December 2018, eligible consecutive adult patients planned for major elective surgery (abdominal and thoracic surgery) were included in the prehabilitation program, without regard to their age. We followed up with patients for 6 months after surgery, regardless of their compliance to the program. Exclusion criteria included pregnancy, an American Society of Anesthesiologists (ASA) physical status score of 4, surgery planned in less than 4 weeks, planned postoperative admission to the intensive care unit, and the patient’s inability to perform the initial physical exam as described below. Other exclusion criteria included patients whose surgery was delayed or cancelled after the initial assessment.

### 2.2. Procedure

As soon as the surgery was planned, a nurse assessed the patients for eligibility, explained the prehabilitation program with its potential benefits and constraints, evaluated their ability to follow the physical program, and performed a G8-questionnaire in patients over 75 years of age [12,13]. If the G8 score was <14 or if any specific reversible disability was detected, a geriatric assessment was planned to confirm the ability to undergo prehabilitation.

If eligible, the first meeting with the prehabilitation team was organized within 48–72 h to assess (i) functional capabilities using a six-minute walk test (6 MWT) and cardiopulmonary exercise testing (CPET), limb mobility, and strength measurement by dynamometry of the dominant and non-dominant hand; (ii) a three-day food survey assessing caloric intake and nutritional balance; and (iii) depressive and anxious elements modifiable with behavioral management using the hospital anxiety and depression scale. Elderly patients were stratified according to Fried’s phenotype, an easy score typically used by anesthesiologists during their evaluation [14], and the short emergency geriatric assessment (SEGA) score that includes two sections: (A) geriatric state with identified risk factors and (B) social environment [15].

At the end of this multidisciplinary assessment, the patient received a personalized prescription combining: (i) home-based physical exercises with both aerobic (moderate-to-intense) and resistance exercises with the intensity of the exercises set at 80% of the maximal heart rate and consisting of either cycling or walking; (ii) dietary advice and a prescription of protein supplements to achieve a protein intake of 1.2 to 1.5 g·kg^−1^·d^−1^ with the indication of consuming 20 g of whey-protein before sleeping on the days of aerobic exercises; and (iii) coping strategies to reduce anxiety [11].

To ensure minimal compliance, patients received a detailed pictorial booklet describing the content of the prescriptions and the instructions to be followed. During this period, they also benefited from a weekly follow-up carried out by telephone by the physiotherapist in charge. 

To allow comparison, identical assessments were organized after four weeks of prehabilitation (before surgery) and six months after surgery.

After the surgery, a postoperative enhanced recovery program was supervised by the same physiotherapists. The medical follow-up was performed with a medical visit at the second month and a similar complete assessment to that previously described after the sixth postoperative month. 

In practice, patients who missed one visit but who continued in the program were defined as absent at the corresponding visit. However, definite interruptions of the program such as early termination or absence at the sixth month resulted in uncompleted data and, therefore, exclusion.

### 2.3. Outcomes

Demographic, surgical, and performance data were collected during the initial visit. 

The main outcome was the change in the 6 MWT over the study period. Secondary variables included the rate of responders after prehabilitation, meaning patients in any group with an improvement in their 6 MWT above 10%. Other variables were quadriceps strength (newtons), maximal power (watts), heart rate reserve (bpm), VO2 at peak (mL·kg^−1^·m^−2^), HAD score, and lean body mass (%). Postoperative length of stay was measured, and postoperative complications were categorized according to the Dindo–Clavien classification [16]. If possible, a reason was given for each premature exit from the study.

### 2.4. Statistical Analysis

#### 2.4.1. Sample Size Calculation

The number of patients to be included was not calculated. As 60 to 80 new patients follow a prehabilitation program every year, we chose to include patients arbitrarily over a period of two and a half years to have enough patients that benefited from a similar procedure.

#### 2.4.2. Statistical Methods 

The results are expressed as numerals (percentage) and median (interquartile range) regardless of their distribution. The comparison between patient groups younger than 65 (young group) and over 65 years of age (elderly group) was based on the Chi-2 or Fisher’s exact tests for categorical data and the Wilcoxon–Mann–Whitney test for continuous data. Performance analysis or patient compliance over time with repeated measures was performed by a linear mixed-effect model. A value of *p* < 0.05 was considered significant. The analysis was performed by SAS v9.4 (Statistical Package for the Social Sciences, IBM; Cary, NC, USA).

## 3. Results

### 3.1. Patients

**Figure 1:**
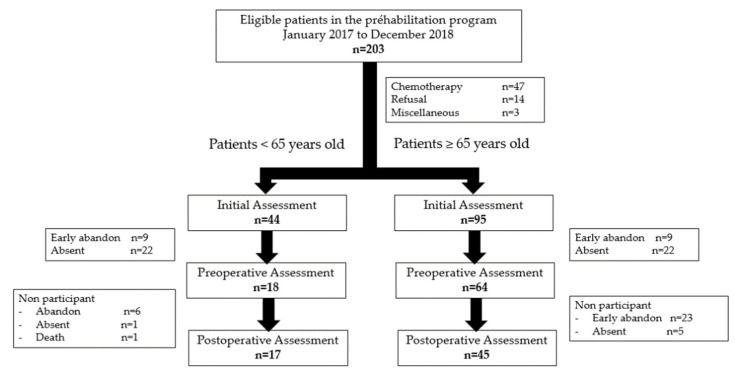
Flow chart.

From January 2017 to June 2019, 139 consecutive patients were included in this program and performed the initial evaluation, with 44 in the younger group (aged below 65 years old) and 95 in the elderly group (Figure 1). Eighteen patients in the younger group (40.9%) and 64 patients (67.4%) in the elderly group completed the preoperative assessment after one month of prehabilitation (*p* = 0.16), while 17 (38.6%) and 45 (47.9%) completed it 6 months after surgery (*p* = 0.62).

### 3.2. Patient Characteristics at Entry

Patient characteristics are presented in Table 1. The median age was 56 (10) years in the young group and 75 (12) years in the elderly group (*p* < 0.001). The Fried frailty scores were similar in both groups, as were the other main comorbidity scores. A relevant difference was found for the SEGA score. Geriatric consultation was necessary for 27 patients, all of them in the elderly group. In this selected group, the G8 score was 14.5 (4).

### 3.3. Outcome Variables over Time

**Figure 2:**
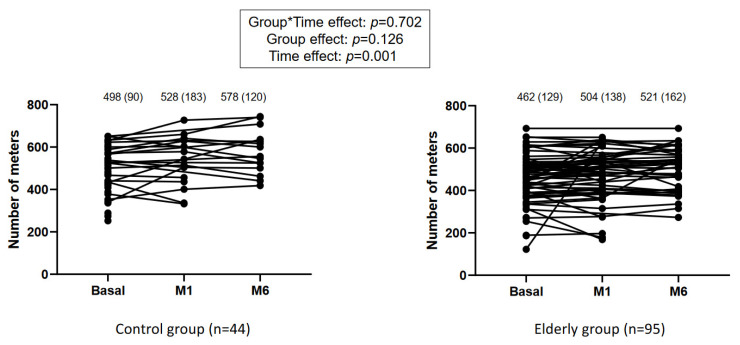
Evolution of the number of steps performed during the 6-min walk test at each time-point. Basal: measure at entry in the study. M1: after one month of prehabilitation. M6: six months after surgery. The number of steps is expressed as median (interquartile range).

Considering the 6 MWT as the primary outcome, statistical analysis revealed no group*time effect (*p* = 0.702) and no group effect (*p* = 0.126), but it revealed a time effect (*p* = 0.001), suggesting its change at each step of the program (Figure 2). Patients in the control group improved the 6 MWT by 80 m (51), while elderly patients improved their performance by 56 m (34) (Figure 2). The rates of response to preoperative prehabilitation were 33% in the young patients and 10% in the elderly patients (*p* = 0.031). This difference was not detected at 6 months with rates of responders of 29% and 20% (*p* = 0.652), respectively.

Table 2 shows changes in other outcome variables over time. Group-time interactions were not statistically different, except for the HADS depression score (*p* = 0.036). The length of stay in intensive or intermediate care units was 0 (2) days vs. 0 (0) days (*p* = 0.53) for the young and elderly, respectively. In the control group, six (13.7%) had more than two postoperative complications according to the Dindo–Clavien classification compared to two (2.1%) in the elderly group (*p* = 0.02). The global rate of complications, irrespective of their range, was 46%. Seven (15.9%) patients in the control group vs. 6 (6.3%) elderly patients were readmitted within 30 days of surgery (*p* = 0.24).

The death rates were similar between the groups at the sixth month with two (4.5%) in the control group and one (1%) in the elderly group (*p* = 0.37) while predicted mortality, evaluated using the POSPOM score, was 22 (11) and 23 (13), respectively, which gave a predicted mortality of 1.73% (Table 1).

## 4. Discussion

Irrespective of the age group, patients improved their functional capabilities after four weeks of a personalized prehabilitation program and maintained this improvement at six months after surgery. More importantly, our study showed significantly increased endurance parameters in the elderly group. Therefore, elderly patients may be considered “responders” to a prehabilitation program as they were able to improve their endurance capacity during prehabilitation and during the follow-up. These results are very encouraging and promising because they demonstrated the importance of including the elderly as early as possible in this program before surgery, assuming an exhaustive evaluation for any existing disability is completed before admittance.

The relevant increase in the 6 MWT at the first month and after six months is novel when compared to previous studies and is similar in both groups after six months (29% in young patients and 20% in elderly patients (*p* = 0.652)). Gillis et al. considered that a difference of 20 m was significant [17]. According to Rasekaba et al., the difference must be more than 56 m between two tests to be considered significant [18]. This is the threshold reached by all patients in our cohort. To explain this answer, two tracks are possible: the initial status and/or the correct response to a personalized training program.

Initially, both groups had similar demographic characteristics including few comorbidities. Patients over 65 years of age had a higher frailty score than the younger group while remaining below eight in the G8 assessment, so they could not be considered frail [14]. In addition, both groups had similar initial functional capabilities. While a theoretical difference would be expected, we can therefore hypothesize that patients over 65 were likely healthy elderly and closer to the under-65 age group. Enright et al. demonstrated a gender-specific regression equation that explained the variance in the distance walked for healthy adults depending on age and BMI: for men, 6 MWT = 1140 m − (5.61 × BMI) − (6.94 × age), and for women, 6 MWT = 1017 m − (6.24 × BMI) − (5.83 × age) [19]. In our cohort, patients under 65 years of age reached 498 m (134) from the initial assessment, and those over 65 years of age reached 462 m (128).

These results support the hypothesis of a close preoperative physical state between the two groups. This favorable physical state may be due to the pre-selection of patients made, on the one hand, by the surgeon and, on the other hand, by the geriatrician. In fact, patients were selected upstream by the surgeons who referred them to either a specialist geriatric consultation or directly to the preoperative nurse consultation.

The responsiveness among the elderly can also be explained by an active care pathway with multimodal customization for the patient. The natural evolution of functional capabilities and endurance parameters during the natural history of cancer shows a constant decrease, especially in the elderly (>80 years old), as demonstrated in a prospective cohort [20]. A common concept is a loss of about 10% of capability after four weeks, and that was not the case in this double cohort with progress in the total distance walked [21]. This personalization of care allows for the overall optimization of the patients and thus improves their future, including the postoperative period [22]. This implication of many actors around the elderly patient was promoted recently as a key to success [23]. Throughout the program, the patients met with all the actors of the program who trained and encouraged them. Dietary and psychological counselling was adapted according to the results obtained. The exercises to be performed at home were also modified, if necessary, to adapt for compliance. After the surgery, the kinetics of recovery were similar in both groups. Improvement of physical abilities preoperatively can be considered as the development of a functional reserve that helps the patient towards enhanced recovery [24]. Indeed, physical exercise allows for better training and a faster response as well as ensuring a patient is more adapted to stress, especially from surgery [25]. Cardiac output increases in response to the effort required. The maximum oxygen consumption increased slightly with an average change of 2.7 mL·kg^−1^·min^−1^ for those under 65 years old and 1.3 mL·kg^−1^·min^−1^ for those over 65 years old (*p* = 0.06). This increase can be considered an increase in the reserve that can be mobilized during intense stress. A threshold below 11 mL·kg^−1^·min^−1^ is predictive of excess mortality [26]. In contrast, a threshold greater than 14 mL·kg^−1^·min^−1^ is a positive predictor of postoperative survival [27]. If the increase was small, the maximum oxygen consumption medians in the cohort were greater than the threshold of 19.3 mL·kg^−1^·min^−1^ (3.9) for the younger patients, and 16.7 mL·kg^−1^·min^−1^ (5.6) for the elderly. In a similar manner, dynamometry increased during prehabilitation and suggested an increase in overall muscle and a decrease in sarcopenic symptoms [28]. Sarcopenia was recently studied in the elderly, showing its impact on the postoperative course or morbidity over time [29]. Interestingly, the frailty phenotype described by Fried et al. included five criteria, three of which are strongly related to muscle: speed gait, handgrip, and ability to carry 5 kg [14].

Another result is the lower incidence of severe postoperative complications in the elderly group even if the length of stay in ICU was similar, with an average duration of two days for both groups. The death rate and readmission were also similar. The application of a prognostic score, such as POSSUM, on this sample suggested a severely ill population, while morbidity was estimated as moderate (23%) and mortality at about 4% [30]. In fact, the results of this study showed a discrepancy between a similar morbidity and a better-than-expected mortality rate. Thus, according to the recent POSPOM score, the predicted mortality of this population was close to 10%, with a median risk score of 32 [30]. The difference in mortality suggests the preventive influence of this personalized pathway before surgery. Minella et al. showed that patients who improved their 6 MWT (33%) had a better postoperative functional recovery, including a significant reduction in the incidence of postoperative complications (2% vs. 18% with *p* = 0.032), in contrast to those who had not progressed (or, for some, had even shown a decline) [13]. Moran et al. reported in a systematic review and meta-analysis that prehabilitation permits a nearly 50% reduction in the incidence of postoperative complications regardless of the type of digestive surgery (OR 0.59, 95% CI (0.38–0.91)) without a significant reduction in the duration of stay [31]. A randomized study of abdominal surgeries found a halving of overall postoperative complications (OR 0.5 (0.3–0.8); *p* = 0.001) and a net decrease in the number of complications per patient (1.4 (1.6) vs. 0.5 (1.0) with *p* = 0.001) [32]. These authors also demonstrated a low risk of patient readmission as well as a medico-economic-favorable analysis [33]. Among the eight patients who developed postoperative complications in our study, this event impaired the postoperative program because only one patient was assessed at the sixth month. Nevertheless, the absolute number of postoperative complications was low, and we did not have a control group to assess the benefit of prehabilitation on the postoperative complications. The certainty of a decrease in complications through prehabilitation has not been established. A recent meta-analysis described a high heterogeneity between studies, which meant it could not make conclusions about outcomes, and, therefore, recommended a specific randomized controlled trial [34].

### Study Limitations

As a limitation, this was a retrospective and monocentric study. Secondly, included patients were carefully selected, especially the elderly, with a double selection (surgeon and geriatrician), and this may explain a healthier population whose evolution closely followed the control group. Exercise at home, as well as during the various sessions, may have been limited by anemia including iron deficiency. Unlikely preoperative hemoglobin determination was not systematic in our practice, and we did not have enough data to investigate this point. Another limitation was the poor rate of good compliance during the entire program, with only one-third of the control group present throughout the program as well as only 41% of the elderly. Finally, there was some overlap between improvement in frailty and improvement in physical activity, especially after orthopedic surgery, but it was not possible to adjust the analysis for the type of surgery due to the limited number of studied patients.

## 5. Conclusions

Prehabilitation benefits patients of all ages. This comparative study found no difference between patients under 65 and those over 65 during the seven months of the (p)rehabilitation program. This result confirms the results of the literature. Adherence and compliance must be improved in view of the decrease in motivation observed postoperatively, especially in the over-65 age group. It appears to be necessary to carry out prospective studies with a control group not benefiting from such a program to study its impact on sarcopenia and postoperative complications, especially delirium and mortality. Programs can be further improved to promote and develop prehabilitation for all patients requiring scheduled surgery.

## Figures and Tables

**Table 1:** Patient characteristics at entry.

		Patients <65 Years Old *n* = 44	Patients ≥65 Years Old *n* = 95	***p***
Age (years)		56 (10)	75 (12)	<0.001
Sex—Female, *n* (%)		23 (52)	42 (44)	0.376
BMI (kg/m²)		22 (9)	25 (7)	0.629
ASA score		2 (1)	2 (1)	0.860
Comorbidities, *n* (%)				
	Myocardial infarction	3 (6.8)	8 (8.4)	1
	Diabetes mellitus	4 (9.1)	12 (12.6)	0.543
	Preoperative anemia	15 (37)	26 (27)	0.680
Type of surgery, *n* (%)				0.779
	Oncologic surgery	34 (77)	58 (61)	
	Abdominal surgery (except oncologic)	1 (2)	9 (9.5)	
	Orthopedic surgery	9 (20)	19 (20)	
Preoperative chemotherapy, *n* (%)		15 (34)	15 (16)	0.015
Preoperative geriatric assessment, *n* (%)		NA	27 (28.4)	NA
G8 assessment		NA	14.5 (4)	NA
Physical performance METS *n* (%)				0.159
	≤3	7 (16)	4 (4)	
	>3 and ≤5	7 (16)	17 (18)	
	>5 and ≤7	24 (55)	60 (63)	
	>7 and ≤9	4 (9)	12 (13)	
	>9	2 (5)	2 (2)	
Biology				
	Hemoglobinemia (g·dL^−1^)	12.0 (2.7)	12.9 (3.8)	0.694
	Albuminemia (g·dL^−1^)	32.0 (8.0)	35.0 (3.0)	0.348
	Creatininemia (µmol·L^−1^)	65.3 (27.0)	77.7 (33.9)	0.003
	Ferritinemia	153.0 (392.0)	124.5 (194.0)	0.316
	B12 Vitamin	262.5 (213.0)	291.0 (308.5)	0.405
	Folates	14 (11.7)	11.8 (15.6)	0.517
Charlson’s score		2 (4.5)	1 (2)	0.308
Fried score		0 (1)	1 (2)	0.060
Frailty according to SEGA scale				
	Part A	2 (3)	3 (3)	0.030
	Part B	0.5 (1)	1 (3)	0.002
Lee’s score		0 (1)	0.5 (1)	0.300
Pospom score		22 (11)	23 (13)	0.891

Results are expressed as numerals (percentage) and median (interquartile range). ASA: American Society of Anesthesiologists; BMI: Body Mass Index; METS: Metabolic Equivalents; NA: Not Applicable.

**Table 2:** Evolution of variables measured repeatedly.

	Patients <65 Years Old *n* = 44	Patients ≥65 Years Old *n* = 95	*p*		
			Group Effect	Time Effect	Group*Time Effect
Quadriceps Strength (NM)			F ratio = 6.45; *p* = 0.012	F ratio = 0.56; *p* = 0.457	F ratio = 2.75; *p* = 0.099
Initial	329 (194)	289 (175)			
Preoperative	343 (155)	298 (130)			
6 Months	401 (209)	305 (115)			
Maximal Power (W)			F ratio = 2.72; *p* = 0.102	F ratio = 18.01; *p* < 0.001	F ratio = 0.02; *p* = 0.880
Initial	81 (40)	77 (45)			
Preoperative	84 (31)	85 (52)			
6 Months	107 (41)	91 (35)			
Heart Rate Reserve (bpm)			F ratio = 4.34; *p* = 0.039	F ratio = 0.47; *p* = 0.492	F ratio = 0.35; *p* = 0.553
Initial	46 (34)	42 (33)			
Preoperative	54 (24)	56 (37)			
6 Months	66 (41)	49 (31)			
Oxygen consumption (mL·kg^−1^·m^−2^)			F ratio = 0.56; *p* = 0.454	F ratio = 19.68; *p* < 0.001	F ratio = 2.51; *p* = 0.116
Initial	15.6 (4.9)	15.1 (5.8)			
Preoperative	19.3 (5.3)	16.6 (5.6)			
6 Months	20.1 (3.2)	20.1 (4.8)			
HAD anxiety			F ratio = 4.76; *p* = 0.031	F ratio = 12.74; *p* = 0.001	F ratio = 0.42; *p* = 0.516
Initial	7 (4.5)	5 (5)			
Preoperative	6 (7)	3.5 (4)			
6 Months	6 (7)	5 (5)			
HAD depression			F ratio = 1.75; *p* = 0.188	F ratio = 0.45; *p* = 0.504	F ratio = 4.46; *p* = 0.036
Initial	5 (6.5)	4 (4)			
Preoperative	3.5 (4)	3 (5)			
6 Months	3 (5)	4 (6)			
Lean mass (%)			F ratio = 0.82; *p* = 0.366	F ratio = 2.38; *p* = 0.126	F ratio = 1.13; *p* = 0.290
Initial	72.9 (16.3)	68.9 (11.9)			
Preoperative	72.9 (20.1)	67.9 (10.2)			
6 Months	77.8 (15.6)	71.0 (13.5)			

Results are expressed as numerals (percentage) and median (interquartile range). HAD anxiety: hospital anxiety scale; HAD depression: hospital depression scale.

## Data Availability

Data are available on the Dryad website open-access repository (doi:10.5061/dryad.rjdfn2zbx).
